# Multicenter analysis and a rapid screening model to predict early novel coronavirus pneumonia using a random forest algorithm

**DOI:** 10.1097/MD.0000000000026279

**Published:** 2021-06-18

**Authors:** Suxia Bao, Hong-yi Pan, Wei Zheng, Qing-Qing Wu, Yi-Ning Dai, Nan-Nan Sun, Tian-Chen Hui, Wen-Hao Wu, Yi-Cheng Huang, Guo-Bo Chen, Qiao-Qiao Yin, Li-Juan Wu, Rong Yan, Ming-Shan Wang, Mei-Juan Chen, Jia-Jie Zhang, Li-Xia Yu, Ji-Chan Shi, Nian Fang, Yue-Fei Shen, Xin-Sheng Xie, Chun-Lian Ma, Wan-Jun Yu, Wen-Hui Tu, Bin Ju, Hai-Jun Huang, Yong-Xi Tong, Hong-Ying Pan

**Affiliations:** a Department of Infectious Diseases, Zhejiang Provincial People's Hospital, People's Hospital of Hangzhou Medical College, Hangzhou 310014; b Department of Internal Medicine, Pujiang people's Hospital, Pujiang 322200; c The Second Clinical Medical College, Zhejiang Chinese Medical University; d Hangzhou Wowjoy Information Technology Co., Ltd, Hangzhou 310000; e Medical College of Qingdao University, Qingdao 266000; f Clinical Research Institute, Zhejiang Provincial People's Hospital, People's Hospital of Hangzhou Medical College, Hangzhou 310014; g Bengbu Medical College, Bengbu 233030; h Department of Infectious Diseases, First People's Hospital of Tongxiang, Jiaxing 100191; i Department of Infectious Diseases, People's Hospital of Shaoxing, Shaoxing 312000; j Department of Infectious Diseases, Wenzhou Central Hospital, Wenzhou 100191; k Department of Infectious Diseases, First Hospital of Taizhou, Taizhou 318020; l Department of Infectious Diseases, First People's Hospital of Xiaoshan, Hangzhou 311200; m Department of Infectious Diseases, First Hospital of Jiaxing, Jiaxing 314000; n Department of Infectious Diseases, First People's Hospital of Wenling, Taizhou 317500; o Department of Respiratory and Critical Care Medicine, Yinzhou People's Hospital, Affiliated Yinzhou Hospital, College of Medicine, Ningbo University, Ningbo 315211; p Department of Infectious Diseases, Taizhou Municipal Hospital, Taizhou 318000, China.

**Keywords:** a rapid screening model, clinical characteristics, multicenter analysis, novel coronavirus pneumonia, random forest algorithm

## Abstract

Early determination of coronavirus disease 2019 (COVID-19) pneumonia from numerous suspected cases is critical for the early isolation and treatment of patients.

The purpose of the study was to develop and validate a rapid screening model to predict early COVID-19 pneumonia from suspected cases using a random forest algorithm in China.

A total of 914 initially suspected COVID-19 pneumonia in multiple centers were prospectively included. The computer-assisted embedding method was used to screen the variables. The random forest algorithm was adopted to build a rapid screening model based on the training set. The screening model was evaluated by the confusion matrix and receiver operating characteristic (ROC) analysis in the validation.

The rapid screening model was set up based on 4 epidemiological features, 3 clinical manifestations, decreased white blood cell count and lymphocytes, and imaging changes on chest X-ray or computed tomography. The area under the ROC curve was 0.956, and the model had a sensitivity of 83.82% and a specificity of 89.57%. The confusion matrix revealed that the prospective screening model had an accuracy of 87.0% for predicting early COVID-19 pneumonia.

Here, we developed and validated a rapid screening model that could predict early COVID-19 pneumonia with high sensitivity and specificity. The use of this model to screen for COVID-19 pneumonia have epidemiological and clinical significance.

## 1 Introduction

The coronavirus disease 2019 (COVID-19) pneumonia outbreak has presented critical challenges for the public health, research, and medical communities. Since December 2019, the COVID-19 pneumonia outbreak has rapidly swept across China and beyond through human-to-human transmission.^[1–3]^ Unlike the improved situation within China, the number of patients with COVID-19 pneumonia in other countries increased swiftly,^[4]^ which means that the battle with COVID-19 is far from over.

Medical reports^[5–7]^ recently showed that the initial symptoms of COVID-19 pneumonia are nonspecific and usually present as fever, cough, headache, vomiting, or diarrhea; additionally, some cases present without any symptoms. Laboratory results can include normal or reduced leukocyte levels and chest computed tomography (CT) findings appearing as patchy/punctate ground-glass opacities in single or multiple lung lobes.^[8]^ Currently, the real-time reverse transcription-polymerase chain reaction (RT-PCR) assessments are regarded as the gold standard for COVID-19 diagnosis.^[9,10]^ Nevertheless, the COVID-19 specific IgM antibody is usually generated 3–5 days after onset, COVID-19 pneumonia cases initially presented negative via RT-PCR.^[11,12]^ There were numerous suspected COVID-19 pneumonia cases in clinics during the COVID-19 infection pandemic season. In addition, a shortage of RT-PCR test kits and specimen sampling uncertainty could adversely impact early COVID-19 pneumonia diagnosis, lead to delayed patient isolation and treatment, and challenge COVID-19 pneumonia epidemiology and prognosis. Therefore, a rapid diagnostic model to screen high-risk or suspected COVID-19 pneumonia patients is urgently needed.

In the research, we aimed to develop and validate a rapid, computer-assisted screening model based on epidemiological data, clinical characteristics, and laboratory results and imaging examinations to detect early COVID-19 pneumonia from numerous suspected subjects. The random forest algorithm was adopted to build a rapid screening model. Model performance was assessed with the receiver operating characteristic curve (ROC) and areas under the curves (AUC). The predictive power of the prediction model was validated by the confusion matrix in the validation set. This is the first prospective screening model to predict early COVID-19 pneumonia, as well as its widespread application in other countries.

## 2 Materials and methods

### 2.1 Study subjects

A total of 914 participants suspected of COVID-19 infection were enrolled from multiple medical institutions in Hangzhou, Shaoxing, Jinxing, Wenzhou, Ningbo, and Taizhou from January 17, 2020 to February 29, 2020. The sample size was calculated by G∗Power 3.1.9.2. The suspected or confirmed patients were diagnosed based on the 7th edition of the Chinese recommendations for the diagnosis and treatment of pneumonia caused by COVID-19. The purpose of the study was to develop and validate a rapid screening model to predict early COVID-19 pneumonia from suspected cases using a random forest algorithm in China.

#### 2.1.1 Suspected COVID-19 pneumonia cases

COVID-19 pneumonia suspected case met any 1 of the epidemiological criteria and any 2 clinical presentation criteria. If there were no epidemiological factors, then the suspected patients should meet 3 of the clinical presentation criteria.

Epidemiological factors: (1) a history of residence or travel in the outbreak area (Wuhan) or its nearby areas, communities with confirmed cases or other areas with persistent local transmission within 14 days; (2) a history of being in contact with confirmed COVID-19 infected patients (positive nucleic acid detection) within 14 days; (3) a history of being in contact with patients with fever or respiratory symptoms who had a history of residence or travel to the outbreak area (Wuhan) or its neighboring areas, communities with confirmed cases or other areas with persistent local transmission within 14 days; and (4) association with a cluster outbreak, defined as the confirmed COVID-19 pneumonia case in a place of work or a family or community, which was not in the outbreak area, within 14 days, along with other patients suffering from a fever or respiratory symptoms.

Clinical presentations: (1) fever and/or respiratory symptoms; (2) typical chest imaging features of COVID-19 pneumonia, such as ground-glass opacities, pulmonary consolidation, and infiltrating shadows; and (3) normal or decreased white blood cells (WBC), or decreased lymphocytes in an early stage of the disease.

#### 2.1.2 Confirmed COVID-19 pneumonia cases

Confirmed COVID-19 pneumonia cases were defined as a suspected COVID-19 pneumonia case that had any 1 of the following criteria: (1) positive COVID-19 nucleic acid found using RT-PCR from sputum, blood samples, throat swab, or stool sample and (2) genetic DNA sequencing results of the samples were homologous with the known COVID-19.

A total of 914 patients were prospectively recruited in the study. The exempt informed consent was approved because the patients would not be exposed to any risks, and the patients’ information was anonymously collected prior to analysis in this observational study. This study was reviewed and approved by the Ethics Committees of all the medical institutions.

### 2.2 Epidemiological factors, clinical characteristics, and laboratory and imaging findings

Epidemiological factors and clinical manifestations were collected for each case. Age, sex, region of residence, body temperature, dry cough, fatigue, dyspnea, sputum, conjunctival congestion, nasal congestion, diarrhea or abdominal ache, dizziness or headache, nausea or vomiting, sore throat, muscle soreness, and comorbidities were recorded for all patients. Blood routine examination and C-reactive protein (CRP) were performed by standard laboratory methods. The X-ray examination followed the common chest protocol: the patients stood with their backs to the X-ray device and their chests pressed firmly against the plate, with their hands resting on the ILIUM, shoulders drooped, upper arms turned inward (pulling apart the shoulder blades), head tilted back slightly, and lower jaw resting on the upper edge of the plate. CT scanning followed the common chest protocol: the patients were placed in a supine position with their arms raised. The patients were instructed to hold their breath during the data acquisition, which included the whole lung volume. The overall scan time was 2 seconds, and the slice thickness for reconstruction was 1.25 mm. Throat swabs, sputum, stool, or blood samples were collected to test for COVID-19 nucleic acid using standardized RT-PCR test kits following the standard protocol. If an initial RT-PCR test was negative, 2 repeated tests were performed after 24 hours and COVID-19 specific IgM and IgG antibodies were negative after 7 days.

### 2.3 Establishment of the rapid screening model

We included age, sex, comorbidities, epidemiological data, clinical symptoms, body temperature, WBC count, lymphocyte count, neutrophil count, and chest imaging findings to establish a novel diagnostic model for COVID-19 pneumonia based on the epidemiology in China. The epidemiological features and symptoms were considered binary variables and were scored as “1” if “yes” and “0” if “no.” The thoracic radiologic findings were simply classified as “normal,” “unilateral local patchy shadowing,” “bilateral multiple ground-glass opacity,” “bilateral diffuse ground-glass shadow with pulmonary consolidation,” and “other imaging alterations such as pulmonary nodule or pleural effusion,” and were scored as “0,” “0.5,” “1,” “2,” and “3,” respectively.

The samples from the patients were classified into a COVID-19 pneumonia group with 361 individuals total and a non-COVID-19 pneumonia group with 553 individuals total, based on the RT-PCR outcomes, which were considered the gold standard; the patients were randomly divided into 2 groups: 80% for model development and 20% for model validation. The computer-assisted embedding method was used to screen the variables. The random forest algorithm was applied to build and validate the predictive model.

### 2.4 Statistical analysis

All statistical analyses were performed using SPSS software (version 19.0, SPSS Inc., IBM, Chicago, IL, USA), G∗Power (version 3.1.9.2), and Scientific Python 3.6 libraries (Scikit-Learn package). Continuous variables are expressed as the mean ± standard deviation and were compared using Student *t* test, and categorical variables are expressed as numbers and percentages and were compared using the chi-squared test. For multiple comparisons, a one-way analysis of variance was applied.

G∗Power was used for sample size, prior analysis was conducted, the effect size was 0.25, α err prob was 0.05, and power was 0.98. The computer-assisted embedding method was used for variable selection, and the algorithm of variable selection used in this study was logistic regression; the threshold of the variable selection was manually set to 0.85 based on the optimal detection principle. Collinearity diagnosis was performed to screen the variables, which were used for all subsequent analyses. In the training set, the random forest algorithm, an ensemble, supervised machine learning algorithm, was used to build a classifier (a predictive model based on a panel), and the importance of each variable was calculated. To evaluate the prediction accuracy of the screening model for determining COVID-19 pneumonia, the predictive model was evaluated by the confusion matrix, ROC, and AUC in the validation set. The cutoff value was defined as the value that allowed the maximum sensitivity and specificity values. All statistical tests were carried out in two-tailed ranges, and a probability level of *P* < 0.05 was considered statistically significant.

### 2.5 Results clinical characteristics

A total of 935 patients were recruited in this study, including 21 excluded patients due to data missing, and 914 participants were eligible for the evaluation. Among them, 553 patients were excluded because of at least 2 negative results by RT-PCR, and the remaining 361 patients were diagnosed as having COVID-19 pneumonia with a positive COVID-19 detection using RT-PCR.

The patient's characteristics are shown in Table 1. Among the 361 COVID-19 pneumonia patients, the mean age was 47.16 ± 14.47 years, and 204 patients were male (56.51%). The mean age of the COVID-19 pneumonia patients was remarkably older than the age of those without COVID-19 pneumonia (*P* < 0.001). Of the confirmed COVID-19 pneumonia patients, 33.24% had a travel or residence history in the outbreak area (Wuhan) within 14 days, 25.76% had contact with patients with fever or respiratory symptoms, and 36.84% were associated with cluster outbreaks within their families or working places.

**Table 1:** Clinical characteristics of the patients with COVID-19 pneumonia/non-COVID-19 pneumonia.

	COVID-19 pneumonia (n = 361)	Non-COVID-19 pneumonia (n = 553)	*P* value
Age (years)	47.16 ± 14.47	37.87 ± 17.94	<0.001
Sex (male)	204 (56.51%)	277 (50.09%)	0.057
Comorbidities	110 (30.47%)	73 (13.20%)	<0.001
Hypertension	71 (19.67%)	42 (7.60%)	<0.001
Diabetes mellitus	23 (6.37%)	14 (2.53%)	0.005
Cancer	4 (1.11%)	5 (0.90%)	0.745
COPD	3 (0.83%)	10 (1.81%)	0.266
Hepatitis B infection	8 (2.22%)	5 (0.90%)	0.151
Others	11^∗^ (3.05%)	14^†^ (2.53%)	0.681
Travel/or residence within 14 days			
The outbreak area (Wuhan)	120 (33.24%)	70 (12.65%)	<0.001
The outbreak area (Wuhan) nearby areas in Hubei Province	9 (2.49%)	68 (12.30%)	<0.001
Other areas with persistent local transmission or communities with confirmed COVID-19 pneumonia cases	162 (44.88%)	279 (50.45%)	0.104
Contacting the patients with fever or respiratory symptoms in 14-days, or who had a history of traveling or residence in the following areas			
The outbreak area (Wuhan)	93 (25.76%)	64 (11.57%)	<0.001
The outbreak area (Wuhan) nearby areas in Hubei Province	8 (2.21%)	24 (4.34%)	0.097
Other areas with persistent local transmission, or communities with confirmed COVID-19 pneumonia cases	99 (27.42%)	81 (14.65%)	<0.001
Association with a cluster outbreak	133 (36.84%)	16 (2.89%)	<0.001
Exposure to wildlife animals	1 (0.27%)	1 (0.18%)	1.000
Contact patients with influenza A	3 (0.83%)	15 (2.71%)	0.052
Contact patients with influenza B	5 (1.38%)	15 (2.71%)	0.248
Fever^‡^	175 (48.48%)	261 (47.20%)	1.000
Body temperature	37.51 ± 0.83	37.48 ± 0.82	0.527
Dry cough (cough without sputum)	158 (43.77%)	214 (38.70%)	0.130
Sputum	130 (36.01%)	136 (24.59%)	<0.001
Fatigue	98 (27.15%)	59 (10.67%)	<0.001
Dyspnea	33 (9.14%)	11 (1.99%)	<0.001
Conjunctival congestion	2 (0.63%)	4 (0.72%)	1.000
Nasal congestion	14 (3.88%)	47 (8.50%)	0.006
Diarrhea or abdominal ache	37 (10.25%)	21 (3.80%)	<0.001
Dizziness or headache	28 (7.76%)	48 (8.68%)	0.628
Nausea or vomiting	13 (3.60%)	7 (1.27%)	0.021
Sore throat	19 (5.26%)	60 (10.85%)	0.004
Muscle soreness	17 (4.71%)	3 (0.54%)	<0.001
White blood cell count (×10^9^)	5.40 ± 2.52	7.30 ± 3.01	<0.001
Normal or reduced white blood cells^§^	340 (94.18%)	459 (83.00%)	<0.001
Lymphocyte count (×10^9^)	1.25 ± 1.03	1.71 ± 0.85	<0.001
Reduced lymphocytes^||^	193 (53.46%)	141 (25.50%)	<0.001
Neutrophil cell count (×10^9^)	3.90 ± 4.28	4.95 ± 3.33	<0.001
Normal or reduced neutrophil cells^¶^	322 (89.20%)	429 (77.58%)	<0.001
C-reactive protein level (mg/L)	20.99 ± 26.71	18.50 ± 35.29	0.254
Chest X-ray or CT scanning			
Normal	16 (4.43%)	242 (43.76%)	<0.001
Unilateral local patchy shadowing	93 (25.76%)	134 (24.23%)	
Bilateral multiple ground-glass opacity	126 (34.90%)	98 (17.72%)	
Bilateral diffuse ground-glass shadowing with pulmonary Consolidation	123 (34.07%)	30 (5.42%)	
Other imaging abnormalities such as pulmonary nodule or pleural effusion	3 (0.88%)	49 (8.86%)	

The most common symptoms of COVID-19 pneumonia were fever (48.48%), dry cough (43.77%), fatigue (27.15%), dyspnea (9.14%), and sputum (36.01%); however, fever was not a specific symptom, as it was also common in non-COVID-19 pneumonia patients (47.20%, *P* = 1.000). Reduced or normal WBC counts were found in 340 (94.18%) of the COVID-19 pneumonia patients, and the mean WBC count was 5.40 ± 2.52 (× 10^9^/L) in the COVID-19 pneumonia group, which was remarkably lower than that in the non-COVID-19 pneumonia group (7.30 ± 3.01, × 10^9^/L, *P* < 0.001). Moreover, reduced lymphocytes were also present in 193 (53.46%) patients; the lymphocytes in COVID-19 pneumonia patients were 1.25 ± 1.03 (×10^9^/L), much lower than those in COVID-19 pneumonia patients (1.71 ± 0.85, × 10^9^/L, *P* < 0.001). No significant difference was found between the 2 groups, in terms of the CRP level (*P* = 0.254). Almost all COVID-19 pneumonia patients (94.37%) had pulmonary radiological abnormalities, such as unilateral or bilateral patchy shadows, stiffening of the lungs, or ground-glass opacity on thoracic X-ray or CT.

### 2.6 Candidate biomarkers associated with early COVID-19 pneumonia

The computer-assisted embedding method was used for variable selection, and this variable selection algorithm applies logic regression in this study. The following 31 variables were adopted for variable selection: age; sex; comorbidities; travel or residence in or near the outbreak area (Wuhan) in Hubei Province, other areas with persistent local transmission, or a community with confirmed COVID-19 pneumonia cases within 14 days; contact with patients with fever or respiratory symptoms from the outbreak area (Wuhan), areas near of the outbreak area (Wuhan) in Hubei Province, other areas with persistent local transmission, or a community with confirmed cases within 14 days; association with a cluster COVID-19 pneumonia outbreak; exposure to wildlife animals; contact with patients with influenza A or influenza B, which were tested by standard kits; the presence of fever, dry cough, sputum, fatigue, dyspnea, conjunctival congestion, nasal congestion, dizziness or headache, nausea or vomiting, sore throat, and muscle soreness; laboratory tests including WBC, lymphocyte, and neutrophil cell counts and CRP levels; and radiological examination findings including chest X-ray or CT scanning.

**Figure 1:**
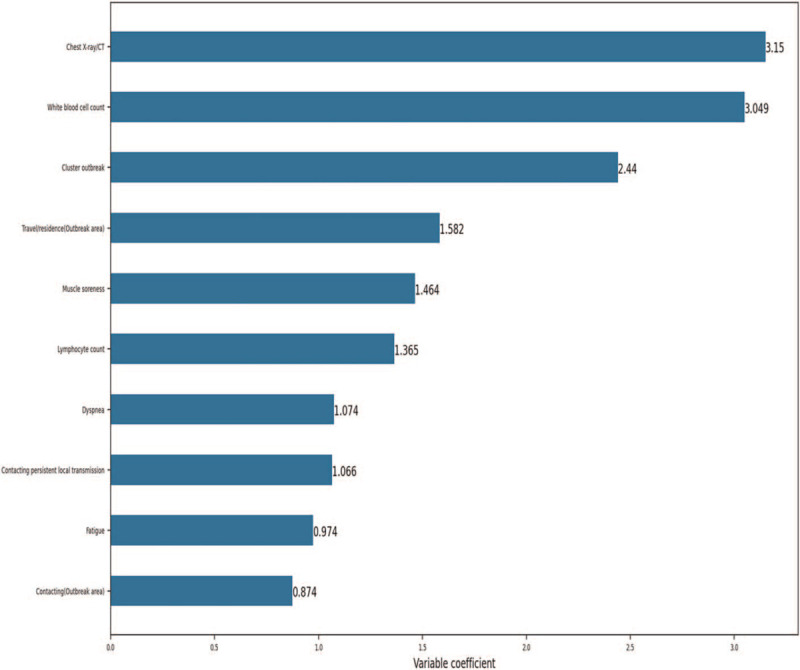
The top 10 variables ranked by regression coefficients. The threshold of variable selection was manually set to an absolute value of 0.85. The top 10 variables were screened for further analysis.

The logistic regression coefficients are shown in Supplementary Table 1, and the variable selection threshold was manually set to be an absolute value of 0.85 based on the optimal detection principle. The top 10 variables ranked by regression coefficient are shown in Fig. 1. These variables were further analyzed for the probability of having COVID-19 pneumonia, including travel or residence history within 14 days in the outbreak area (Wuhan); contact with patients with fever or respiratory symptoms within 14 days who had a travel or residence history in the outbreak area (Wuhan); contact with patients from other areas with persistent local transmission or community with confirmed cases; association with a cluster outbreak; the presence of fatigue, dyspnea, and muscle soreness, reduced WBC and lymphocytes; and imaging findings on chest radiography. In addition, all the tolerances of the top 10 variables were >0.1, and all the variance inflation factors were <10. Therefore, no collinearity was found among the variables (Table 2).

**Table 2:** Collinearity statistics among the top 10 variables ranked by regression coefficient.

Parameter	Tolerance	VIF
Chest X-ray or CT	0.42	2.38
Cluster outbreak	0.78	1.29
Travel/residence (Wuhan)	0.75	1.33
Contacting with others (Wuhan)	0.75	1.34
Contact with others (other areas)	0.75	1.34
Muscle soreness	0.94	1.06
Dyspnea	0.90	1.11
Fatigue	0.79	1.26
Lymphocyte count	0.25	4.02
White blood cell count	0.23	4.41

### 2.7 Development and validation of a model to predict the probability of early COVID-19 pneumonia

**Figure 2:**
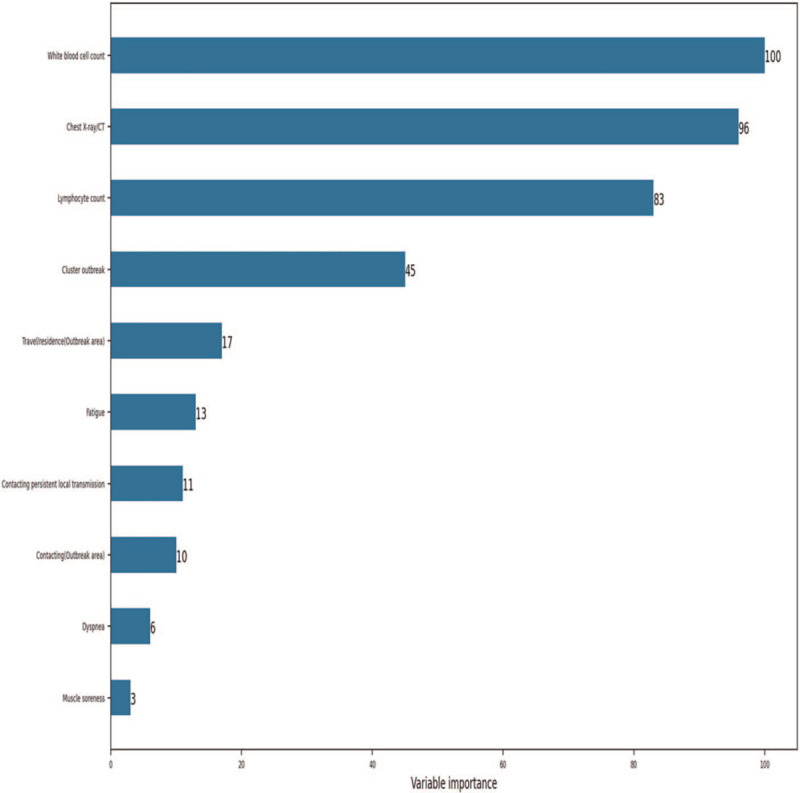
The importance of the predictor variables in differentiating the probability of early COVID-19 pneumonia in the random forest model. The relative measure was scaled to a maximum of 100 and used to compare the importance of the variables in the model. COVID-19 = coronavirus disease 2019.

To identify crucial predictors for the probability of early COVID-19 pneumonia, a random forest model was trained on 80% of the patients using the top 10 variables. We established the importance ranking with the random forest algorithm. High ranks with variable importance are important for tree building and prediction. As shown in Fig. 2, imaging findings on chest radiography, reduced WBC and lymphocytes, and association with a cluster outbreak were the most important predictors in the random forest model with n_estimators = 40 and criterion = entropy.

**Figure 3:**
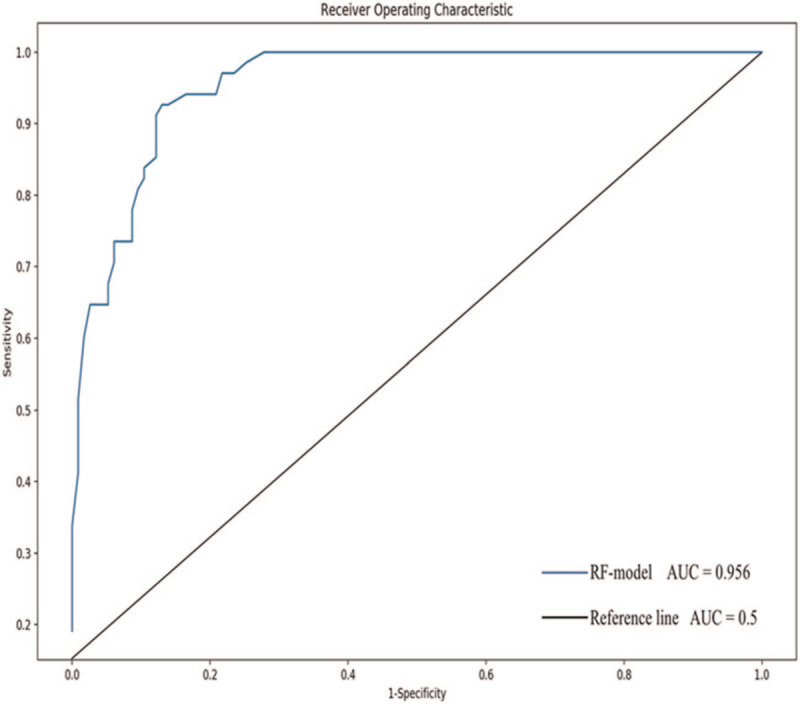
The capacity of the screening variables to predict early COVID-19 pneumonia. The ROC curve and AUC in the rapid screening model for discriminating the probability of early COVID-19 pneumonia. AUC = area under the curve, COVID-19 pneumonia = novel coronavirus pneumonia, ROC = receiver operating characteristic curve.

Model performance was assessed on the 20% validation set with ROC and AUC. As shown in Table 3, the AUC was the highest with an n_estimators = 40, which was therefore selected as the best value. These findings indicated that the AUC was 0.956 with a sensitivity = 83.82% and a specificity = 89.57% to predict the probability of COVID-19 pneumonia (Fig. 3).

**Table 3:** Diagnostic sensitivity, specificity and AUC of the fast screening model with different parameters.

n_estimators	Sensitivity	Specificity	AUC
1	0.7058	0.8522	0.779
5	0.8676	0.8609	0.9426
10	0.8235	0.8783	0.9491
20	0.8235	0.8783	0.9493
30	0.8235	0.8783	0.9527
40	0.8382	0.8957	0.9555
50	0.8235	0.8957	0.9552
60	0.8088	0.8957	0.9529
70	0.8382	0.8957	0.9525
80	0.8382	0.9043	0.9519
90	0.8382	0.8957	0.9525
100	0.8529	0.8783	0.9516

The predictive power of the prediction model for COVID-19 pneumonia was also subsequently validated by the confusion matrix in the validation set (Table 4). The overall predictive percentage was 87.4%. The classified predictive percentages were 83.8% and 89.6% in COVID-19 pneumonia and non-COVID-19 pneumonia patients, respectively.

**Table 4:** The confusion matrix in predicting early COVID-19 pneumonia in the validation set.

Parameter	Group size	COVID-19 pneumonia	Non-COVID-19 pneumonia	Precision	Recall	F1-score
COVID-19 pneumonia	68	57	11	0.83	0.84	0.83
Non-COVID-19 pneumonia	115	12	103	0.90	0.90	0.90
Overall				0.87	0.87	0.87

## 3 Discussion

In this study, we analyzed and compared the epidemiological factors, clinical manifestations, and laboratory and imaging findings between COVID-19 pneumonia patients and suspected COVID-19 pneumonia patients, for whom COVID-19 pneumonia was excluded. We applied the computer-assisted embedding method and random forest algorithm to build and validate a predictive model for early COVID-19 pneumonia. The model included using 4 epidemiological features: travel or residence in the outbreak area (Wuhan); contact with patients with fever or respiratory symptoms from the outbreak area (Wuhan) within 14 days; contact with patients from other areas with persistent local transmission or communities with confirmed cases; and association with a cluster outbreak. The model also included 3 clinical manifestations: fatigue, dyspnea, and muscle soreness; decreased WBC and lymphocyte count; and imaging changes on chest X-ray or CT scanning. The diagnostic performance of this established model was excellent, with an AUC of 0.956. The confusion matrix showed that the overall predictive percentage was 87.4%.

Prior studies have shown that most COVID-19 pneumonia patients were characterized by non-specific clinical presentations, such as fever, cough, fatigue, myalgia, diarrhea, nausea, headache, and sore throat in the early stage of the disease.^[13]^ A proportion of patients gradually progressed to dyspnea, especially in patients with low immune functions.^[12]^ During treatment, the clinicians found that dyspnea was more likely to occur in populations with low immune functions than in those with normal immune functions.^[6]^ The onset of complications, such as arrhythmia, acute respiratory distress syndrome, and shock was often a sign of poor prognosis.^[14–16]^

Among the laboratory results, the most common laboratory abnormalities were leukopenia and lymphocytopenia. Previous study showed that hypoalbuminemia elevated CRP and lactate dehydrogenase, and decreased CD8 count could be found in some patients as well.^[13]^ The most frequent imaging findings were patchy/punctate ground-glass opacities in a single lobe or multiple lobes of the lungs.^[17]^ In addition, abnormalities on chest CT scanning could reflect disease progress and severity.^[18]^ COVID-19 infection could also present with normal pulmonary imaging, particularly in the early stage, suggesting a necessity to combine epidemiology with clinical characteristics, laboratory tests, and imaging findings in the screening and diagnosis of COVID-19 pneumonia.^[19]^

RT-PCR can provide confirmation of the diagnosis of COVID-19 pneumonia.^[9]^ RT-PCR has high specificity and sensitivity and has been widely used in determining different coronavirus infections; however, The RT-PCR has some disadvantages, such as time-consuming, test kit shortages, and specimen sampling issues. Furthermore, RT-PCR might show false-negative results when used with unstable kits or non-standardized sampling, and repeated tests are required for a number of patients with initial negative RT-PCR results.^[20]^ Moreover, COVID-19 specific IgM antibodies are usually generated 3–5 days after onset. Some COVID-19 pneumonia patients were diagnosed only based on clinical and imaging findings due to earlier negative testing results for viral RNA in the outbreak area. All of these presentations and disadvantages have made early COVID-19 pneumonia diagnosis challenging, especially during the pandemic, and have prevented timely isolation and early treatment. A rapid screening diagnostic model is urgently needed to distinguish highly suspicious patients in a large scale population to support epidemiologists and help clinicians make early treatment decisions to ultimately reduce patient mortality.

We applied the random forest algorithm as a classification method that consists of multiple nodes of decision trees in establishing a concise and accurate diagnosis model. This method has clear advantages including a low chance of overfit, more robust noise reduction, faster training speed, and more prediction accuracy.^[21]^ Moreover, compared with other prediction models, this model could more effectively identify interactions and nonlinear relationships between variables.^[22]^

There were 10 candidate variables selected by the computer-assisted embedding method in this study. We set the random forest parameters to “n_estimators = 40 and criterion = entropy” and calculated the importance of each variable according to the original random-forest algorithm. The rapid screening model was subsequently established based on 4 epidemiological features, 3 clinical characteristics, 2 laboratory test results, and radiographic imaging findings. The area under the ROC curve was 0.956 with a high sensitivity of 83.82% and a high specificity of 89.57%, and the confusion matrix achieved 87.0% accuracy for the screening model to predict early COVID-19 pneumonia.

There were some limitations in this study. First, the patients enrolled were limited to China, which may lead to certain regional restrictions, in particular in the epidemiology of possible compromises in different regions. Global studies are needed to assess the application of the model. Second, our research was confined to early and rapid screening, with no adequate information supported on this disease progression and prognosis. Follow-up studies are necessary. Third, we screened only some critical predictors to fit the model and the overall fit of the model could be affected under the limitation of sample size and predictors.

In summary, the use of this screening model to rapidly distinguish COVID-19 pneumonia patients from a large scale of suspected patients has great significance in both epidemiology and clinical therapeutics under the circumstance of continuing widespread COVID-19 infection. Unlike the methods of virus isolation, RT-PCR, or specific IgM antibody assays, this early screening model is economical, uncomplicated, and fast, and it may save some medical resources and life by reducing COVID-19 pneumonia mortalities regionally or globally.

## Acknowledgments

We would like to thank all the patients and staff at the multicenter hospitals involved in this study.

## Author contributions

**Conceptualization:** Hongyi Pan, Wei Zheng, Yining Dai.

**Data curation:** Wei Zheng, Qingqing Wu, Qiaoqiao Yin.

**Formal analysis:** Hongyi Pan, Wei Zheng, Qingqing Wu, Yining Dai, Nannan Sun.

**Funding acquisition:** Yining Dai, Hongying Pan.

**Investigation:** Suxia Bao, Tianchen Hui, Wenhao Wu, Yicheng Huang, Guobo Chen, Qiaoqiao Yin, Rong Yan, Mingshan Wang, Meijuan Chen, Jiajie Zhang, Lixia Yu, Jichan Shi, Nian Fang, Yuefei Shen, Xinsheng Xie, Chunlian Ma, Wanjun Wu, Wenhui Tu, Bin Ju, Haijun Huang, Yongxi Tong, Hongying Pan.

**Methodology:** Suxia Bao, Hongyi Pan, Nannan Sun, Tianchen Hui, Wenhao Wu, Yicheng Huang, Guobo Chen, Qiaoqiao Yin, Rong Yan, Mingshan Wang, Meijuan Chen, Jiajie Zhang, Lixia Yu, Jichan Shi, Nian Fang, Xinsheng Xie, Chunlian Ma, Wanjun Wu, Wenhui Tu, Bin Ju, Haijun Huang, Yongxi Tong, Hongying Pan.

**Project administration:** Xinsheng Xie, Chunlian Ma.

**Resources:** Yicheng Huang, Guobo Chen, Lixia Yu, Yuefei Shen.

**Software:** Suxia Bao, Hongyi Pan, Lijuan Wu, Rong Yan, Mingshan Wang, Bin Ju, Hongying Pan.

**Supervision:** Suxia Bao, Lijuan Wu, Rong Yan, Jiajie Zhang.

**Validation:** Lijuan Wu, Rong Yan, Nian Fang.

**Visualization:** Mingshan Wang, Meijuan Chen, Jiajie Zhang, Jichan Shi.

**Writing – original draft:** Suxia Bao.

**Writing – review & editing:** Haijun Huang, Yongxi Tong, Hongying Pan.

## Supplementary Material

Supplemental Digital Content
