# Supplementary material for: Multicenter analysis and a rapid screening model to predict early novel coronavirus pneumonia using a random forest algorithm
Source: Medicine (Baltimore). 2021 Jun 18;100(24):e26279. doi: 10.1097/MD.0000000000026279 (PMC8213313; doi:10.1097/MD.0000000000026279)
Supplement: Supplemental Digital Content [file medi-100-e26279-s001.docx]

**Supplementary Table 1.** The logistic regression coefficients for candidate biomarkers associated with early COVID-19 pneumonia.

| **Parameter** | **Coefficient** |
| --- | --- |
| Chest X-ray or CT | 3.150419645 |
| Cluster outbreak | 2.440019628 |
| Travel or residence (Wuhan) | 1.581914598 |
| Muscle soreness | 1.464095479 |
| Dyspnea | 1.074132193 |
| Contact with patients with fever or respiratory symptoms from other areas with persistent local transmission | 1.066091792 |
| Fatigue | 0.973701816 |
| Contact with patients with fever or respiratory symptoms from the outbreak area (Wuhan) | 0.874007934 |
| Diarrhea or stomachache | 0.832667779 |
| Comorbidities | 0.680917658 |
| Travel or residence from other areas with persistent local transmission | 0.269534674 |
| Dry cough | 0.261972136 |
| Sex | 0.20159605 |
| Sputum | 0.181238729 |
| Contact with patients with influenza A | 0.14015106 |
| Contact with patients from neighboring areas of Outbreak area (Wuhan) in Hubei Province | 0.069954271 |
| Nausea or vomiting | 0.053086069 |
| Dizziness or headache | 0.003202162 |
| Contact with patients with suspected fever or respiratory symptoms | -0.065428366 |
| Age | -0.177060286 |
| Body temperature | -0.189969581 |
| C-reactive protein level | -0.190468476 |
| Exposure to wildlife | -0.220917061 |
| Conjunctival congestion | -0.280595888 |
| Contact with patients with influenza B | -0.352641264 |
| Nasal congestion | -0.520009866 |
| Neutrophil cell count | -0.540651515 |
| Sore throat | -0.662410912 |
| Travel or residence in neighboring areas of Outbreak area (Wuhan) in Hubei Province | -0.733024197 |
| Lymphocyte count | -1.364978582 |
| White blood cell count | -3.049360345 |
